# Addressing Lupus Health Disparities: The MONARCAS Community and Academic Collaborative Program

**DOI:** 10.1089/heq.2017.0060

**Published:** 2018-07-01

**Authors:** Karen Mancera-Cuevas, Patricia Canessa, Joan S. Chmiel, Elizabeth A. Hahn, Anh H. Chung, Daniel L. Erickson, Rosalind Ramsey-Goldman

**Affiliations:** ^1^ Division of Rheumatology, Northwestern University Feinberg School of Medicine, Chicago, Illinois.; ^2^ Department of Health Equity, Illinois Public Health Association, Springfield, Illinois.; ^3^ Department of Preventive Medicine, Northwestern University Feinberg School of Medicine, Chicago, Illinois.; ^4^ Department of Medical Social Sciences and Center for Patient-Centered Outcomes, Northwestern University Feinberg School of Medicine, Chicago, Illinois.

**Keywords:** curriculum, language, Latino, lupus, Popular Opinion Leader (POL)

## Abstract

**Purpose:** The Centers for Disease Control (CDC) Popular Opinion Leader (POL) model was implemented in a lupus education program (MONARCAS) for the Latino community. The program aim was to increase lupus awareness by training high school students, community health workers, and parents.

**Methods:** A curriculum was developed training POLs to disseminate concepts about lupus signs and symptoms. Pre- and post-program questions assessed lupus knowledge and message dissemination.

**Results:** POL groups represented distinct demographic characteristics with Spanish or English language dominance. POLs reported increased lupus knowledge and program satisfaction.

**Conclusions:** Future program goals should aim to increase understanding and improving access to care for Latino communities affected by lupus.

## Introduction

Lupus is an autoimmune disease with varying levels of medical complexity. Symptoms include hair loss, skin rashes, arthritis, cardiovascular, pulmonary, body swelling, or cognitive challenges.^1^ In the United States, 1.5 million individuals have been diagnosed with lupus, and the disease is more common in racial and ethnic minorities.^1^ Research indicates that timely access to healthcare is important in improving detection and prognosis of diagnosed lupus.^2^ Lupus is an emerging challenge for Latinos and the multifactorial reasons are often attributed to delayed or missed diagnoses, lack of awareness, fragmented care, limited access to subspecialty care, inadequate social support, and difficulties with assimilation and acculturation in Latino communities.^3^ Early interventions are recommended to reduce disease burden among diverse communities to help improve quality of life.^4^

Lupus has an earlier onset, more severe disease course, and increased morbidity and mortality in Latinos compared with whites.^5^ There is greater mortality from lupus in Latinos who live below the poverty line and endure fragmentation of care due to complexity of the disease.^5^ The Grupo Latino Americano De Estudio del Lupus (GLADEL) Multinational Latin American Prospective Cohort of 1214 patients with lupus, described ethnic and disease heterogeneity among Latinos, and documented worse outcomes among mestizos compared with whites.^6^

The “MONARCAS” program was developed to address health disparities in the Mexican Pilsen community in Chicago, Illinois by raising awareness of lupus in the community. “MONARCAS” describes the butterfly that migrates annually between Mexico and the U.S. heartland and is the shape of a facial rash found in persons with lupus. The MONARCAS program utilizes a U.S. Centers for Disease Control (CDC)-adapted Popular Opinion Leader (POL) model to engage social networks, stakeholders, and communities in educational initiatives about the significance of lupus through casual conversations initiated by local opinion leaders.^7^ The POL model engages communities in conversation to facilitate dissemination of a health message in targeted areas through adaptation of social network theory, which stresses the importance of connections or relations for understanding health.^8^ The objectives of the current study were to examine (1) pre- and post-program knowledge of lupus among the POLs, and (2) geographic patterns of message dissemination by POLs.

## Methods

**FIG. 1.**
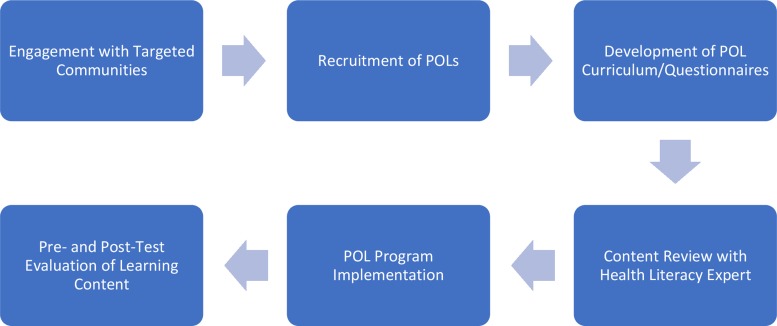
POL Program Flowchart describes steps in the formulation of the POL lupus program implementation. POL, Popular Opinion Leader.

Study procedures were considered exempt by the Northwestern University Institutional Review Board due to scope of the project. Figure 1 describes POL program development and implementation. Meetings were held with the Community Field Director (FD) whose role was to oversee recruitment, education, and follow-up engagement with POLs in the targeted communities. The FD coordinated replication of the POL model in new communities with local leaders. English and Spanish-speaking POLs were recruited for participation in the dissemination effort over a 6-month period with a partner targeting candidates pertaining to community stakeholder groups. The POL cohorts were engaged an average of 3 months after recruitment so the FD could ensure POL model fidelity. A curriculum was developed before the POLs were recruited to instruct on becoming a POL in the community and disseminate lupus education including signs and symptoms of the disease. Questionnaires were first developed in English and then translated into Spanish^9^ early in the process to assess core concepts appropriate for trainees' varied learning levels. The questionnaires were translated by a bilingual expert to ensure that forms were equivalent within the appropriate context into Spanish language for persons preferring to answer questions in their native language. Before implementation and dissemination, a health literacy expert reviewed educational materials and assessments.

All materials were designed for a 5th grade learning level so that POLs with elementary school education could be recruited. The program utilized pre- and post-test questionnaires on learning content before and after curriculum administration over a 2-week timeframe in three POL groups who included a mixture of predominantly English and Spanish speakers from Chicago-area community settings. The pre- and post-test questionnaires, which covered lupus knowledge, ethnicity, migration, education, and assimilation/acculturation were distributed during the 2-week session timeframe and participants completed the questionnaires on paper. Assimilation/acculturation was measured with questionnaires covering English-language dominance; preference in reading/speaking; ethnic pride; and years in the United States. POL participants were trained in community research methods including collection of addresses where information would be delivered.

Descriptive statistics were used to summarize characteristics of the POLs in the communities and their responses, and to investigate how assimilation and lupus fact acquisition may be associated with lupus knowledge. Lupus message dissemination was assessed through address information provided by the POLs and Geographic Information System (GIS) mapping was used to summarize message dissemination patterns for each POL group. Statistical comparisons of post- versus pre-test correct response rates for each question, for each POL group, were done using chi-square tests.

## Results

Three distinct POL groups represented three mutually exclusive groups of varying ages and demographics: 13 high school students participating in a youth program; 14 community health workers (CHWs) trained in chronic disease management and referral; and 28 parents who attended weekly meetings in a Latino-serving neighborhood school. Each POL group represented different Latino groups: the Students were English-speaking second-generation Mexican American, the CHW group was from racial ethnic backgrounds including the Caribbean and Central/South America, and the parents were entirely first-generation Mexican. The majority of CHWs and parents came to the United States after age 2 years, although exact age of migration from country of origin was not measured.

No POL participant withdrew from the study, but two participants from the parent group did not complete all of the questionnaires as they were unable to stay during the post-test portion of the session. The students self-described as equally fluent in English and Spanish language. Table 1 describes responses from the basic data form. The CHW group varied in degrees of Spanish language dominance, the Parent group was more Spanish-language dominant, and the Student group reported predominantly English and Spanish language equal dominance. This language dominance difference did not impact delivery of the curriculum as it was delivered similarly in the English and Spanish-language community settings. Despite language dominance differences, across all participant groups there was 77% agreement on the importance of belonging to the Latino ethnic group and having ethnic pride based on self-reported responses. Thus, the roles of language and culture intersected despite influences of acculturation in three participant groups. Additionally, although the majority of CHW (64%) and parents (89%) spoke and read Spanish, fewer in these groups reported reading and writing in Spanish, that is, CHW (36%) and parents (46%), respectively. This may be reflective of the overlap in the reading criteria language dominance in both questions. A weakness in all three groups was the low number of community stakeholder contacts, particularly in a study design leveraging the capability of the individual to be influential within social networks.

**Table 1.** **Characteristics of the Three Popular Opinion Leader Groups**

Characteristics	CHW (*n*=14) *n* (%)	Students (*n*=13) *n* (%)	Parents (*n*=28) *n* (%)
Birthplace			
Mexico	9 (64)	2 (15)	28 (100)
United States	2 (14)	11 (85)	
Other	3 (21)		
Moved to the United States after age 2	12 (86)	10 (77)	26 (92)
Resided in the United States ≥5 years	10 (71)	13 (100)	28 (100)
Previously attended school	14 (100)	12 (92)	27 (96)
What language do you mostly speak & read? (%)	9 (64) (Spanish)	11 (85) (Spanish and English equally)	25 (89) (Spanish)
What language do you mostly read & write? (%)	5 (36) (Spanish)	11 (85) (Spanish and English equally)	13 (46) (Spanish)
Do you have a strong sense of belonging to your ethnic group? (% yes)	10 (71)	10 (77)	23 (82)
Do you have community stakeholder contacts? (% yes)	4 (29)	0 (0)	4 (14)

Descriptors classify responses provided by POL participants in all three groups. Entries in the table represent the number (percentage) of respondents.

CHW, community health worker.

**FIG. 2.**
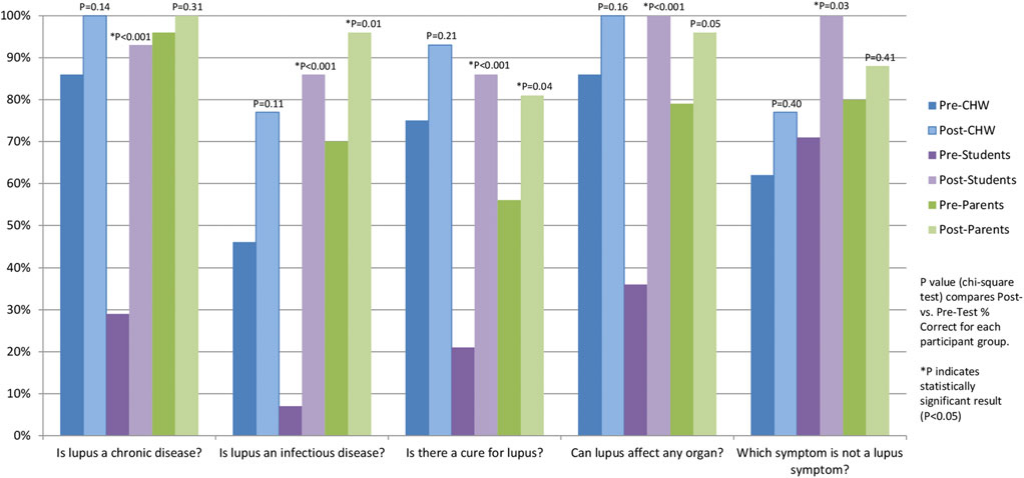
Lupus knowledge questions. Figure shows the percentage of correct answers to each question on the pre- and post-test questionnaires for each POL group.

Figure 2 summarizes pre- and post-test correct responses for lupus knowledge questions. Participants demonstrated greater understanding of lupus topics between the pre- and post-questionnaire intervals with all lupus questions. The students in particular demonstrated significant lupus knowledge improvement from pre- to post-test on all questions (*p*<0.05). The CHWs had greater knowledge gain on the post-test although these differences were not statistically significant. The Parents group correct responses varied depending on the question, for example, they knew about lupus but not as much detail about the disease (*p*≤0.05 on three of five questions) such as understanding that lupus is a chronic disease, and distinguishing which symptom is not a lupus symptom.

**FIG. 3.**
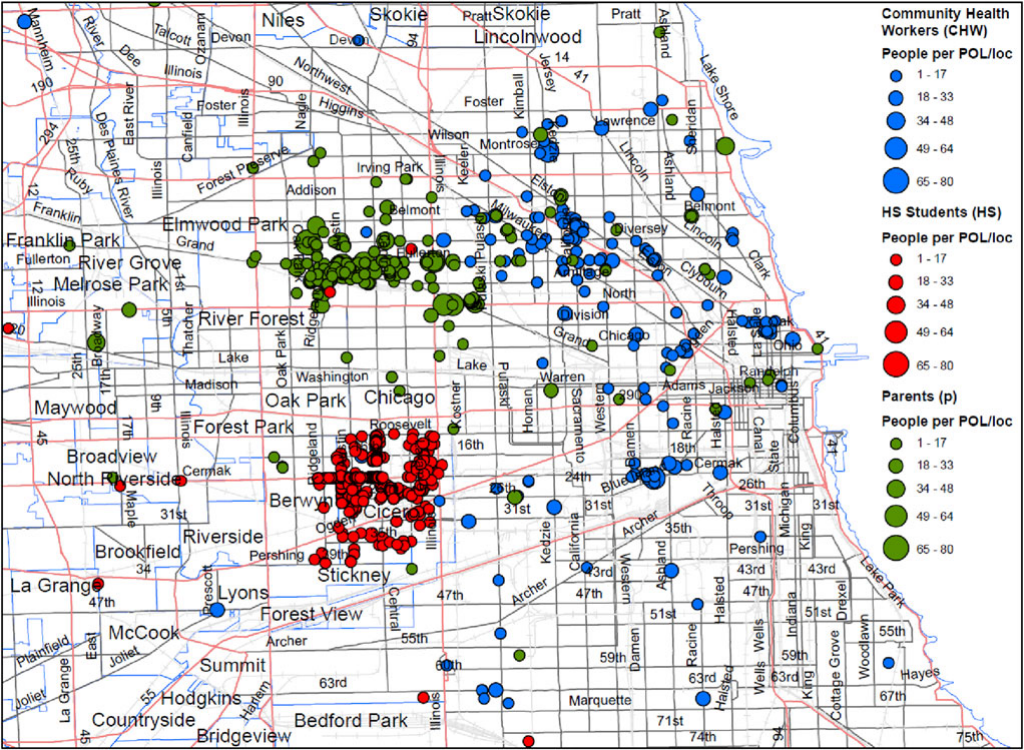
GIS-targeted Chicago areas displaying the educational dissemination scope of activity of the three POL groups: CHW, Student, and Parent (GIS output distinguishes the three POL groups dissemination of their messages). As shown in the GIS map, the students were largely concentrated in the community of Cicero. The Parent group covered more geographic territory outside of the Northwest side of Chicago and the CHWs distributed information in the South and Southwest suburbs. The GIS map was utilized to measure lupus awareness saturation in a community and each dot reflects degree of POL involvement in the targeted communities. In theory, POLs with larger social networks were able to create greater impact over a 3-month engagement period. The study expectation was that each POL would engage in conversations with at least 50 individuals. CHW, community health worker; GIS, Geographic Information System.

As shown in the Figure 3 GIS map, the students were largely concentrated in the community of Cicero. The Parent group covered more geographic territory outside of the Northwest side of Chicago and the CHWs distributed information in the South and Southwest suburbs. The GIS map was utilized to measure lupus awareness saturation in a community and each dot reflects degree of POL involvement in the targeted communities. In theory, POLs with larger social networks were able to create greater impact over a 3-month engagement period. The study expectation was that each POL would engage in conversations with at least 50 individuals.

## Summary and Discussion

All groups acquired lupus knowledge between the pre- and post-test evaluation, as demonstrated by the improvement in the percentages of correct answers between pre- and post-tests. The students showed the greatest significant progress with regard to educational attainment of POL curriculum lupus knowledge. As a group, the youth demonstrated enthusiasm and eagerness to participate and disseminate the lupus awareness message in their community. For the students, positive youth development enabling components of self-esteem, self-efficacy, and hopefulness also help protect against risks of secondary negative health behaviors existing in urban settings.^10^ Participation in the POL program could help youth with learning skills that include being healthy throughout adolescence, developing healthy behaviors for a lifetime, and learning how to access and use the healthcare system.^11^

Regarding English and Spanish-language proficiency, overall language capability was not tested but was required for the communication method used in the program to properly adhere to the design of the POL model. Comfort with the dominant language is associated with the capability to speak and perceive.^12^ In the case of the POLs, primary language reported is Spanish in two of the three POL groups. Because of variability in language proficiency, bilingual individuals may also have different levels of proficiency in written comprehension.^13^ Because of the demand of processing language, bilingual individuals must then effectively process instructions^14^ and in other cases back translate to the primary language.^15^ This poses unique challenges for the POLs in this study because the majority were bilingual and may have experienced challenges understanding curriculum content or the pre-/post-test questionnaire content.

Our findings suggest potential for further development of the program, however, limitations due to small sample size and literacy between groups make comparisons between groups challenging. Nonetheless, our data suggest that knowledge increased in all three participant groups. Importantly, the Student group demonstrated the greatest increase in lupus knowledge between the pre- versus post-test periods. Future goals are to teach the POLs the importance of impactful message outreach through GIS mapping, and to more effectively tailor training programs based on the POL training group profile (i.e., CHW or Students) to capitalize on group strengths for message dissemination.

## Abbreviations Used

CDC: Centers for Disease Control
CHW: community health worker
FD: Field Director
GIS: Geographic Information System
GLADEL: Grupo Latino Americano De Estudio del Lupus
POL: Popular Opinion Leader

## References

[1] Lupus Foundation of America. Lupus Facts. 2017 Available at www.lupus.org Accessed 1015, 2017

[2] Drenkard C, Bao G, Dennis G, et al. Burden of systemic lupus erythematosus on employment and work productivity: data from a large cohort in the southeastern United States. Arthritis Care Res. 2014;66:878–887 10.1002/acr.22245 24339382

[3] Contreras G, Lenz O, Pardo V, et al. Outcomes in African-Americans and Hispanics with lupus nephritis. Kidney Int. 2006;69:1846–1851 16598205 10.1038/sj.ki.5000243

[4] Demas K, Costenbader K Disparities in lupus care and outcomes. Curr Opin Rheumatol. 2009;21:102–109 19339919 10.1097/BOR.0b013e328323daad PMC2774141

[5] Alarcon G, McGwin G, Bastian H, et al. Systemic lupus erythematosus in three ethnic group VII: predictors of early mortality in the LUMINA cohort. Arthritis Rheum. 2001;45:191–202 11324784 10.1002/1529-0131(200104)45:2<191::AID-ANR173>3.0.CO;2-2

[6] Pons-Estel B, Catoggio L, Cardiel M, et al. The GLADEL multinational Latin American prospective inception cohort of 1,214 patients with systemic lupus erythematosus: ethnic and disease heterogeneity among “Hispanics”. Medicine. 2004;83:1–17 14747764 10.1097/01.md.0000104742.42401.e2

[7] Kelly J, Murphy D, Sikkema K, et al. Randomised, controlled, community-level HIV-prevention intervention for sexual-risk behaviour among homosexual men in US cities. Community HIV Prevention Research Collaborative. Lancet. 1997;350:1500–1505 9388397 10.1016/s0140-6736(97)07439-4

[8] Valente T Social Networks and Health: Models, Methods, and Applications. New York: Oxford University Press, 2010

[9] Maneesriwongul W, Dixon J Instrument translation process: a methods review. J Adv Nurs. 2004;48:175–186 15369498 10.1111/j.1365-2648.2004.03185.x

[10] Allen M, Rosas-Lee M, Ortega L, et al. They just respect you for who you are: contributors to educator positive youth development promotion for Somali, Latino, and Hmong Students. J Prim Prev. 2016;37:71–86 26740113 10.1007/s10935-015-0415-2 PMC6121717

[11] Banspach S, Zaza S, Dittus P, et al. CDC Grand Rounds: adolescence-Preparing for lifelong health and wellness. MMWR Morb Mortal Wkly Rep. 2016;65:759–762 27491062 10.15585/mmwr.mm6530a2

[12] Kopke B Neurolinguistics aspects of attrition. J Neurolinguistics. 2004;17:3–30

[13] Edmonds A Correlates and cross-linguistic comparisons of informativeness and efficiency on Nicholas and Brookshire discourse stimuli in Spanish/English bilingual adults. J Speech Lang Hear Res. 2013;56:1298–1313 23785179 10.1044/1092-4388(2012/12-0065)

[14] Paradis J, Genessee F Syntactic acquisition in bilingual children. Autonomous or independent? Stud Second Lang Acquis. 1996;18:1–25

[15] Leather J Second-language speech research: an introduction. Lang Learn. 1999;49:1–56

